# Prognostic and clinicopathological significance of SIRT1 expression in NSCLC: a meta-analysis

**DOI:** 10.18632/oncotarget.19244

**Published:** 2017-07-12

**Authors:** Yifei Chen, Tao Wang, Wei Wang, Jiahao Hu, Ruiting Li, Shaojun He, Jiong Yang

**Affiliations:** ^1^ Department of Respiratory and Critical Care Medicine, Zhongnan Hospital of Wuhan University, Wuhan, Hubei 430071, P. R. China; ^2^ Department of Respiratory and Critical Care Medicine, Renmin Hospital of Wuhan University, Wuhan, Hubei 430060, P. R. China

**Keywords:** NSCLC, SIRT1, overall survival, prognosis, meta-analysis

## Abstract

Non-small cell lung cancer (NSCLC) is the most common type of lung cancer. The prognosis of NSCLC is extremely poor and it is urgently to find a new marker. Numerous studies have confirmed that silent mating type information regulation 2 homolog-1 (sirtuin1; SIRT1) is abnormally expressed in NSCLC. This meta-analysis was performed to investigate the prognostic and clinicopathological significance of SIRT1 in NSCLC. A total of seven eligible studies, including 6 on clinicopathological features, 7 on prognosis were identified from the databases. Pooled hazard ratios (HRs) or odds ratios (OR) and 95% confidence intervals (95% CIs) were calculated using random- or fixed-effects models. Results revealed that high expression of SIRT1 was associated with poor overall survival in NSCLC patients (HR=1.99, 95% CI: 1.33-2.98, *P*=0.0009). Moreover, SIRT1 were related to histological grade (OR= 2.00, 95% CI= 1.05–3.78, *P*= 0.02) of NSCLC patients. In conclusion, our present meta-analysis indicated that SIRT1 may serve as a promising marker for prognosis of patients with NSCLC.

## INTRODUCTION

Lung cancer is a common malignant cancer worldwide, which makes up about 18.2% of all malignant tumor mortality in the world [1, 2]. It is estimated that 222,500 new cases will be diagnosed with lung cancer and caused about 155,870 people’s death according to the cancer statistics 2017 [3]. Non-small cell lung cancer (NSCLC), which consists predominantly of squamous cell carcinoma (SCC) and adenocarcinoma (AD), is the most common type of lung cancer [4]. Despite surgical resection, systemic chemotherapy and targeted drugs can treat lung cancer patients successfully, the prognosis of NSCLC still remains poor owing to the late diagnosis of patients [5, 6]. Therefore, it is of great importance to discover markers specific for lung cancer in order to provide better individualized treatment.

Silent mating-type information regulation 2 homologue 1 (sirtuin1; SIRT1) is the closest mammalian homologue of yeast Sir2 and is an NAD-dependent protein deacetylase, which regulates life span in accordance with nutritional provisions under conditions of stress [7]. It is reported that SIRT1 played important roles in many biological processes, including aging, DNA repair, apoptosis and cell senescence [8–10]. Nowadays, many studies demonstrated that aberrant expression of SIRT1 was found in various cancers, containing hepatocellular carcinoma [11, 12], gastric carcinoma [13], breast carcinoma [14] and colorectal carcinoma [15, 16], indicating that SIRT1plays important roles in the initial and progress of cancers.

To date, abundant researches have confirmed that SIRT1 was abnormally expressed in NSCLC. The overexpression of SIRT1 is significantly correlated with high pathological T-stage and lymph node metastasis in NSCLC [17]. SIRT1 increases vessel density through facilitating endothelial cell branching and proliferation and promotes lung tumor growth through down-regulation of DLL4/Notch signaling and deacetylation of Notch1 intracellular domain [18], the Inactivation of SIRT1 can exert its antitumor activities through the tumor suppressor p27 in NSCLC [19]. Moreover, SIRT1 could contribute to the development of lung cancer through TNF-α/β-catenin axis [20]. Recent studies have revealed that abnormally expressed SIRT1was associated with prognosis of NSCLC [17, 21–26]. Therefore, a hypothetical that there might be a significant correlation between the expression of SIRT1 and the clinical outcomes of NSCLC was speculated.

With the aim to evaluate the association between the SIRT1 expression and NSCLC clinical outcomes comprehensively, we carried out this meta-analysis of the published articles to explore the clinical value of SIRT1 in NSCLC.

## RESULTS

### Study selection and characteristics

**Figure 1:**
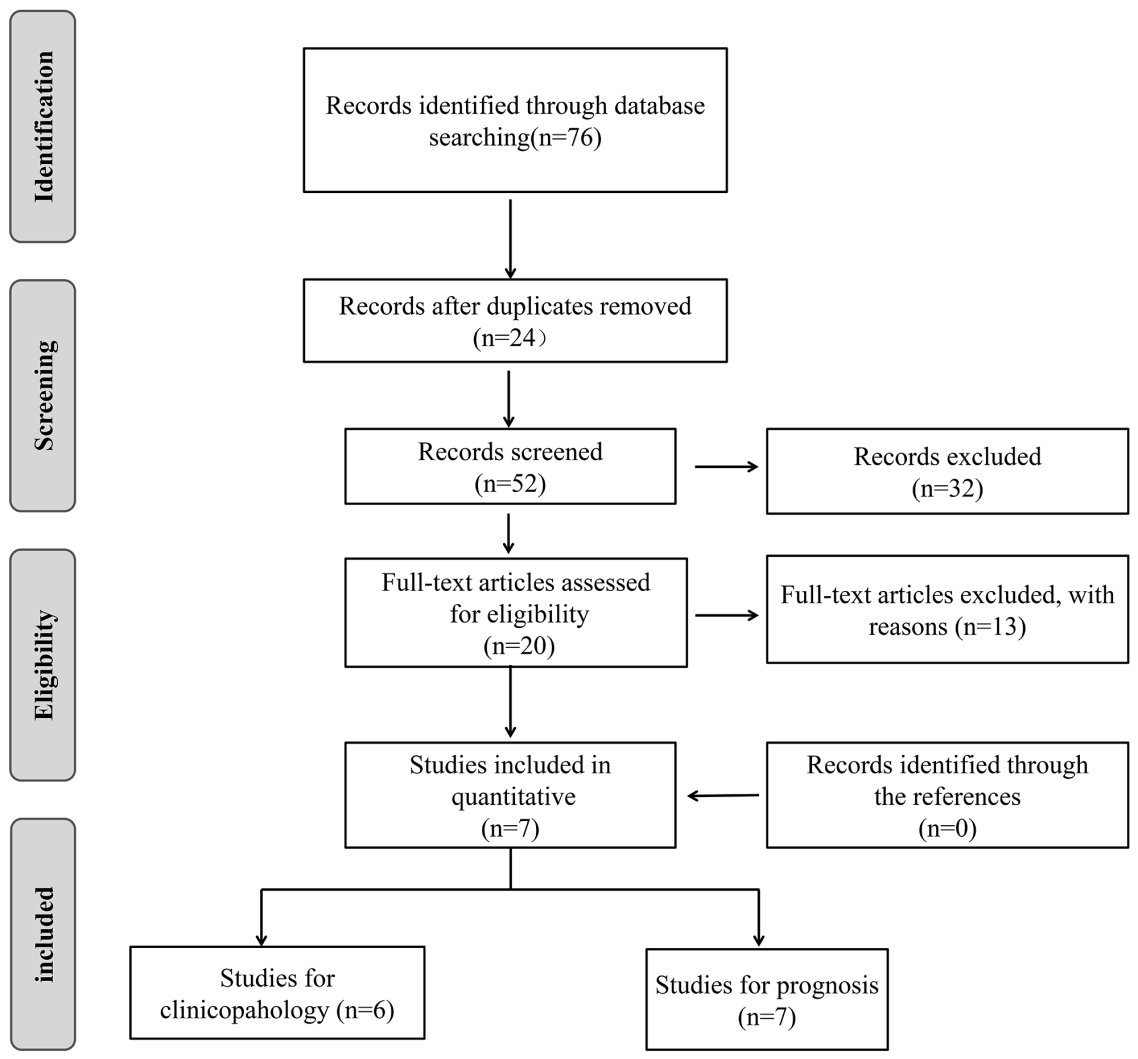
Flow diagram of study search and selection process

Figure 1 showed the selection process of included studies. The electronic search identified 76 records from PubMed, EMBASE, and Web of Science and 24 articles were excluded due to duplicated publication. After we screened the titles and abstracts, 32 irrelevant articles were excluded. Subsequently, the 20 remaining full-text articles were assessed and 13 studies were further excluded according to the exclusion criteria. Finally, seven articles were included in the current meta-analysis [17, 21, 22, 24–27]. All the included studies used FFPE tissue to detect the expression of SIRT1. As shown in Table 1, the studies were published from 2013 to 2016, and included 979 patients with the highest number of 295 and the smallest number of 40. All the studies used immunolytic enzyme histochemistry technology to evaluate the expression level of SIRT1. Among the seven studies, three are from China [17, 25, 27], one from Japan [24], one from USA [21], one from Iran [22] and one from Spain [26].

**Table 1:** Characteristics of the included studies in this meta-analysis

Author	Year	Country	Total number	Histologic grade (W and M/Poorly)	Method	Type of survival data	Follow up time (month)	HR	NOS score
**Noh**	2013	USA	144	105/39	IHC	OS	NR	Reported	7
**Gharabaghi**	2016	Iran	40	22/18	IHC	OS	50	Reported	7
**Lin**	2016	Japan	260	61/199	IHC	OS	125	Reported	8
**Li**	2015	China	75	-	IHC	OS	120	Reported	8
**Zhang**	2013	China	295	242/53	IHC	OS	25	Reported	7
**Grbesa**	2015	Spain	105	42/57	IHC	OS	144	Survival Curve	8
**Wang**	2016	China	60	43/17	IHC	OS	100	Reported	7

IHC: immunohistochemistry; HR: hazard ratio; W, well; M, moderate

### Prognostic value of SIRT1 expession in NSCLC

**Figure 2:**
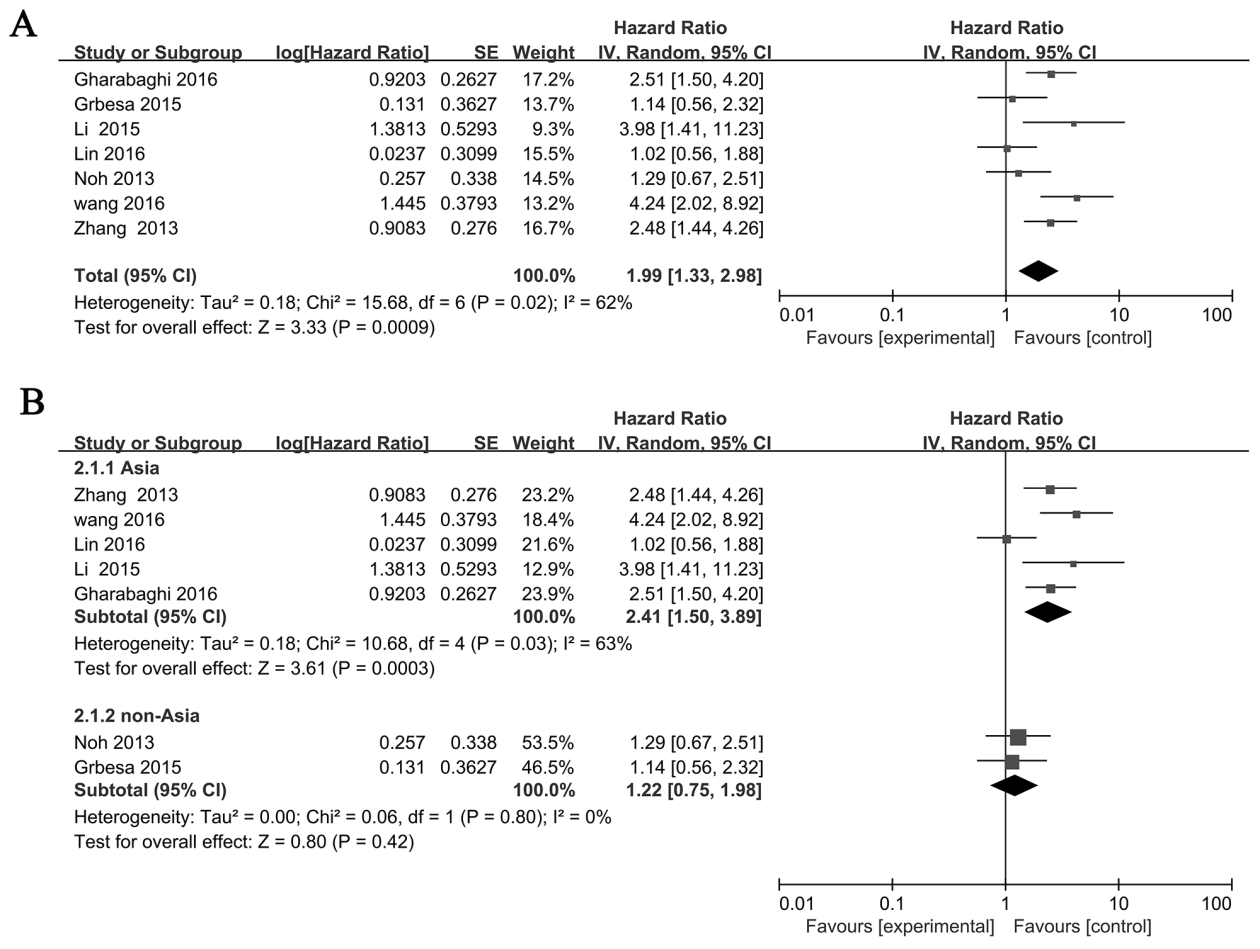
Forest plot of HRs for the association of SIRT1 expression in NSCLC with OS **(A)** All NSCLC patients, **(B)** Asian and non-Asian NSCLC patients. The point estimate is bounded by a 95% confidence interval, and the perpendicular line represents no increased risk for the outcome. NSCLC: non-small cell lung cancer.

There are 7 studies [17, 21, 22, 24–27] investigated the relationship betweenSIRT1 expression and overall survival (OS) with a total number of 979 NSCLC patients. We then analyzed the association between the expression of the SIRT1 and patient survival. The pooled HR for OS showed that SIRT1 was associated with poor prognosis in NSCLC patients (HR=1.99, 95% CI: 1.33-2.98, *P*=0.0009 random-effects) (Figure 2A). Subsequent analyses of subgroup based on Asian and non-Asian were performed. Result revealed that SIRT1 expression was significantly related to OS in Asian (HR= 2.41, 95% CI=1.50-3.89, *P*<0.01, random-effect), but not in non-Asian (HR=1.22, 95% CI=0.75-1.98, *P*=0.42, random-effect). (Figure 2B).

### Association between SIRT1 and histological grade

**Figure 3:**
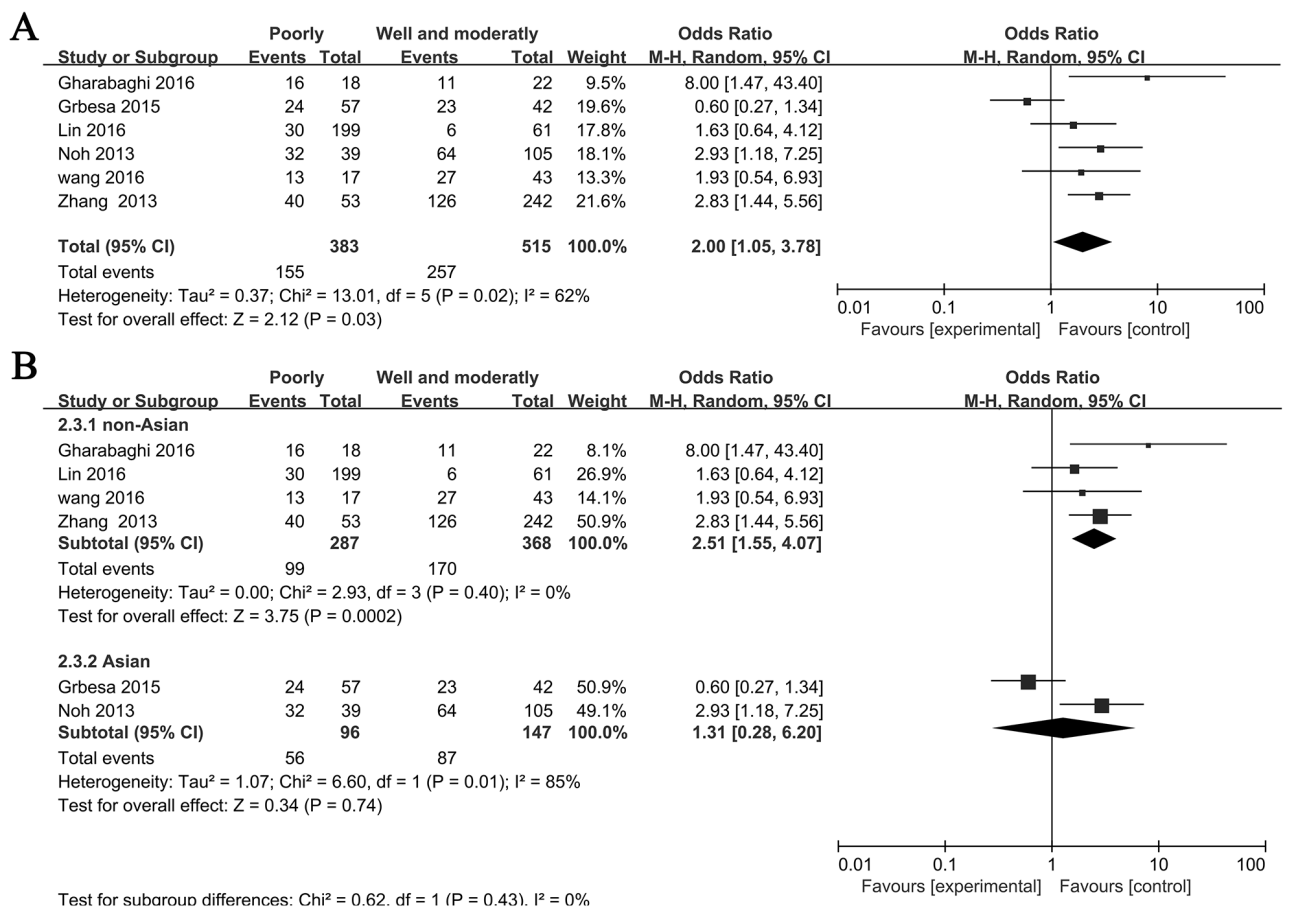
Forest plot for association between SIRT1 and histological grade **(A)** All NSCLC patients, **(B)** Asian and non-Asian NSCLC patients.

Six studies investigated the correlation between the expression of SIRT1 and histological grade, so we explored whether an association between SIRT1 expression and histological grade was existed. The pooled OR from 383 poorly and 515 well and moderately differented is shown in Figure 3A (OR= 2.00, 95% CI= 1.05–3.78, *P*= 0.03, random-effects). Subgroup analyses were done based on on Asian and non-Asian. We found that SIRT1 expression was more frequent in patients that are poorly differented in Asian (OR= 2.51, 95% CI= 1.55–4.07, *P*<0.01) but not tend to be frequent in non-Asian (OR= 1.31, 95% CI= 0.28–6.20, *P*= 0.74) (Figure 3B).

### Sensitivity analyses and publication bias

**Figure 4:**
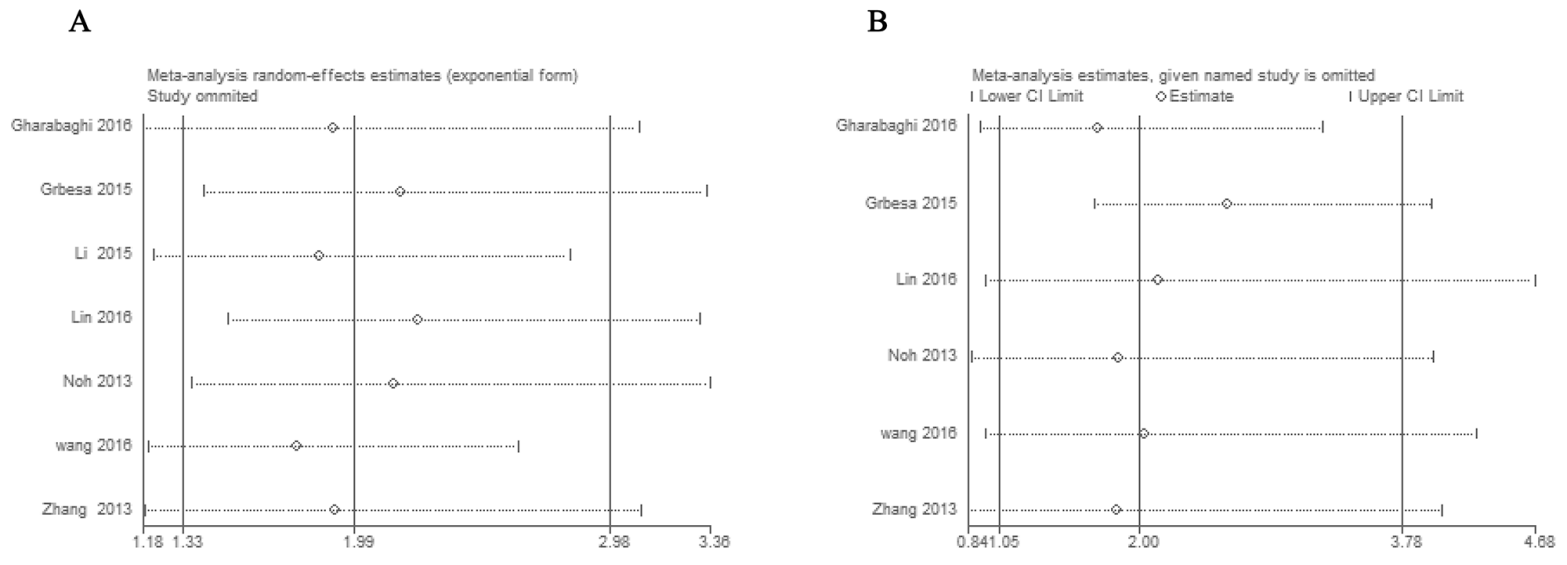
Sensitivity analyses of the studies **(A)** Overall survival, **(B)** histological grade.

**Figure 5:**
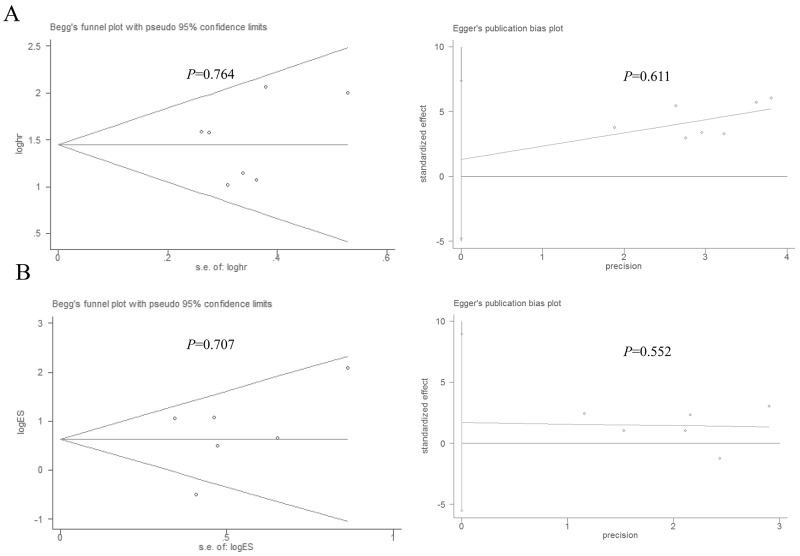
Begg’s and Egger’s test for publication bias **(A)** Overall survival, **(B)** histological grade.

Stata11.0 software was used to perform sensitivity analysis to assess the stability of our results. The results indicated that individual study had little influence on our final results, which validated the stablity and crediblity of our results (Figure 4). Both Begg’s test and Egger’s test were used to evaluate the publication bias. The results indicated no publication bias existed in all subgroups since all the values of *P* > 0.05 (Figure 5).

## DISCUSSION

NSCLC is one of the leading causes of cancer-related death worldwide [28]. The overall prognosis of NSCLC is poor and it has a high risk of recurrence after surgical resection [29]. Thus it is urgently needed to identify new biomarkers for NSCLC. SIRT1, homologue of the yeast Sir2 protein, is the most researched sirtuin in the mammalian sirtuin family [30]. It has been commonly accepted that SIRT1 plays critical roles in many important biological processes such as cell proliferation, cell death, senescence and stress response [26]. Numrous studies reported the involvement of SIRT1 in NSCLC [31–33]. Xie *et al.* showed that overexpression of SIRT1 in transgenic mice facilitated endothelial cell branching, thus increasing vessel density and lung tumor growth [18]. Cha *et al.* [34] found that celecoxib and sulindac can inhibit TGF-β1-induced EMT and suppress lung cancer cell migration and invasion via downregulation of SIRT1. In addition, aberant expresion of SIRT1 was reported to be associated with clinical and progress of NSCLC patients.

The role of SIRT1 in NSCLC is debatable. A large number of studies have reported that SIRT1 was high expressed in NSCLC. For exmple, SIRT1 was reported to be a tumor promoter in lung adenocarcinoma [23] and can enhance β-catenin accumulation then to activate TNF-α transcription, leading to sustained inflammation and lung cancer development [20]. On the other hand, SIRT1 activation has been reported to hamper lung cancer metastasis [33] and Beane *et al.* [35] validated that the activity of SIRT1 was down-regulated in tumors from smokers. Owing to the controversial role of SIRT1 in NSCLC, it is of great value to perform this meta-analysis of the current literature to gain a better understanding between SIRT1 and NSCLC.

In the present meta-analysis, we found that the increased expressions of SIRT1 were associated with poor prognosis, which is in consistent with four of the included studies. However, the remaning three articles showed no corelation betwwen SIRT1 and OS of NSCLC. When we did subgroup analysis, we found that SIRT1 expression was related to OS in Asian rather than in non-Asian, indicating that the discrepency may be due to different origin of the samples. Then, we explored the relation between SIRT1 expression and histological grade. We found that SIRT1 tend to be highly expressed in poorly differented NSCLC patients, indicating a tumorgenesis role of SIRT1 in NSCLC.

It should be noted that there were limitations in our analysis. Firstly, the definition of SIRT1 positive expression varied between the selected studies, which may reduce the reliability of our study; secondly, since only English language papers were included, the data collection may be incomplete; thirdly, the HR value from 1 study was got from KaplaneMeier survival curve instead of the given data, which may generate inaccurate results; fourthly, some studies distinguished the expression location of SIRT1 but somes didn’t, which may reduce the credibility of the study; fifthly, the sample size maybe too small to fully confirm the association between SIRT1 and clinicopathological characteristics, which needs more studies. Despite the limitations mentioned above, our study is the first meta-analysis to explore the association between SIRT1 expression and clinical features of NSCLC. Importantly, we utilized a statistical approach to combine the results of multiple studies, achieving an objective result on the basis of strict inclusion and exclusion criteria.

In summary, our study was the first to evaluate the correlation between SIRT1 and clinical values of patients with NSCLC. We revealed that SIRT1 expression was related to histological grade. Besides, we found that SIRT1 can be used as potential prognostic markers for NSCLC. However, considering the limitations of individual study, in future, large-scale and and good quality studies were needed to confirm our results.

## MATERIALS AND METHODS

### Search strategy

PubMed, EMBASE, and Web of Science were systematically searched in February 2017 using the search terms: “SIRT1”, “silent mating type information regulation 2 homolog-1”, “sirtuin 1” and “NSCLC”, “non-small cell lung cancer“, “LAD”, “lung adenocarcinoma”, “LSCC”, “lung squamous cell cancer”. Additionally, references of retrieved relevant articles were screened to identify potentially eligible literature. We did not limit our search by country, race and date.

### Inclusion and exclusion criteria

Inclusion criteria: According to the principle of PICO (participant, intervention, comparison and outcomes) [36], studies were selected for the following criteria:

• Participants: patients diagnosed with NSCLC.
• Intervention: SIRT1 positive or highly expressed.
• Comparison: SIRT1 negative or low expressed.
• Outcomes: shorten in overall survival time, histological grade reviewed as poorly.

The exclusion criteria included: (1) studies without usable data; (2) duplicate publications; (3) sample cases fewer than 30; (4) reviews, letters, single case reports; (5) animal studies.

### Data extraction and quality assessment

Two investigators extracted the data from eligible studies independently and the following information was collected: name of the first author, year of publication, number of cases, histopathological grade of tumors (moderately and well vs poorly), method, information about OS (HR value and follow-up duration). In our study, we defined OS as duration of time from Starting from randomization to death caused by any reason. Data for the characteristics of the studies were summarized and organized into a table format.

Newcastle-Ottawa Scale score (NOS) was used to assess the quality of the studies. The scale allocates a maximum of nine points for the quality of selection, comparability, exposure, and outcomes for study participants. The total scores ranged from 0 to 9, and a study with a score of 7 or more represents a good quality [37].

### Statistical analysis

All analyses were conducted using the STATA software version 11.0 (Stata Corporation, College Station, Texas, USA) or Review Manager Version 5.3. HR and 95% CIs were used to assess the association between SIRT1expresion and OS in NSCLC, and ORs and 95% CIs were used to evaluate the relation between SIRT1 expression and clinical features. When the *I*^2^≤50%, the fixed effect model was used, otherwise, a random effect model was applied [38]. HRs with their 95% CIs were got directly from data in articles or from Kaplan–Meier survival curves using Engauge Digitizer version 4.1 [39]. Sensitivity analysis was conducted by omitting the study sequentially. Begg’s and Egger’s test were used for assessing the publication bias. Two-sided *P* < 0.05 was considered statistically significant.
